# Trends in prevalence and number of cases of diagnosed type 2 diabetes in Germany: Projections until 2050

**DOI:** 10.25646/13381

**Published:** 2025-09-17

**Authors:** Jens Baumert, Lukas Reitzle, Ralph Brinks, Ronny Kuhnert, Christin Heidemann

**Affiliations:** 1 Robert Koch Institute, Department of Epidemiology and Health Monitoring, Berlin, Germany; 2 Witten/Herdecke University, Faculty of Health (Department of Human Medicine), Chair of Medical Biometry and Epidemiology (MBE), Witten, Germany

**Keywords:** Type 2 diabetes, Prevalence, Prognosis, Incidence, Mortality

## Abstract

**Background:**

In order to assess the prevention and care needs for type 2 diabetes in the coming decades from a public health perspective, forecasts on the trends in prevalence and case numbers are essential.

**Methods:**

The data are based on age-specific estimates of diagnosed diabetes prevalence from the survey German Health Update (GEDA) 2022, and on the proportion of type 2 diabetes derived from routine health insurance data. Using routine data on the incidence and excess mortality of diabetes, various scenarios for the future trends of type 2 diabetes are modelled using an illness-death model.

**Results:**

Based on a type 2 diabetes prevalence of 8.6 % in 2022 (women: 8.2 %, men: 9.2 %), corresponding to a total of 6.05 million cases (women: 2.92 million, men 3.13 million), the prevalence is expected to rise to 16.1 % by 2050 (women: 14.8 %, men: 17.4 %), with the number of cases increasing to 11.01 million (women: 5.19 million, men: 5.82 million). Assuming a 2.0 % annual decline in incidence, the prevalence is expected to rise to only 12.2 % (8.39 million cases); with a simultaneous 2.0 % annual decline in excess mortality, the prevalence is expected to reach 13.0 % (8.94 million cases).

**Conclusions:**

The prognosis is mainly influenced by changes in incidence. Primary preventive approaches to reduce risk factors for type 2 diabetes are therefore crucial to counteract an increase in the number of type 2 diabetes cases.

## 1. Introduction

Diabetes mellitus comprises a group of chronic metabolic diseases characterised by elevated blood sugar levels [1]. Type 2 diabetes is the most common form of diabetes, accounting for over 90 % of all diabetes cases, and mainly manifests itself in middle or older adulthood [2]. The care of type 2 diabetes, along with its complications and comorbidities are associated with high costs [3, 4]. Behavioural risk factors (e.g. poor diet and lack of exercise, obesity) and adverse environmental and social conditions (e.g. air pollution, social deprivation) can contribute to the development of type 2 diabetes [5, 6]. Since these factors are considered potentially preventable or modifiable, there are opportunities for strategies to prevent the onset of type 2 diabetes.

In Germany, the prevalence of medically diagnosed diabetes in 2022 is around 10 % among people aged 18 and over, based on the nationwide survey German Health Update (GEDA); women have a lower prevalence than men (9.2 % vs. 11.1 %) [7]. Similar prevalence rates have been observed based on routine data from people with statutory health insurance [8, 9]. In recent decades, the prevalence of diabetes significantly increased, both in Germany (2003 – 2022, age-standardised to the 2013 European standard population: women 6.2 %–8.3 %, men 5.8 %–10.9 %) [7] and worldwide (1990–2022, age-standardised to the World Health Organization (WHO) standard population: women 6.3 %–14.9 %, men 5.8 %–14.6 %) [10], which can be explained primarily by the increase in the prevalence of type 2 diabetes [11]. Due to demographic changes (ageing population) as well as developments in the number of new cases (incidence) and mortality of type 2 diabetes, a further increase in the prevalence of type 2 diabetes has been projected for Germany [12 – 15]. In order to assess the need for prevention and care services for type 2 diabetes, as well as to plan appropriate public health measures or adapt them if necessary, predictions of trends in the incidence of type 2 diabetes in the coming decades are essential.

Based on routine data from recent years, the incidence of type 2 diabetes has been declining in Germany, but this trend did not continue at the same rate during the pandemic years [16 – 18]. Two studies observed a significant decline in incidence in 2020, followed by a resurgence in 2021 and 2022 to the levels seen in 2015 and 2014, respectively [16, 18]. Another study found no significant difference in incidence rates in the 2020 to 2021 pandemic phase compared to the 2012 to 2019 pre-pandemic phase, but did observe a less pronounced decline in incidence in the 2018 to 2021 period compared to the 2012 to 2017 period [17]. In principle, a decrease in the incidence of diagnosed type 2 diabetes could be attributed to improvements in primary prevention and the resulting reduction in the above-mentioned risk factors. On the other hand, an increase in incidence could indicate an adverse development of risk factors, but also improved early detection. The change in incidence patterns during the pandemic may be related to indirect and direct consequences of the pandemic, such as an initial reduction in medical consultations due to infection control measures and thus a reduction in the diagnosis of new cases, followed by a catch-up effect in consultations and diagnoses [16, 19], as well as a possible SARS-CoV-2-related increase in the risk of developing type 2 diabetes [20, 21]. Continued evaluation of incidence is necessary to assess post-pandemic incidence trends.

There are only isolated data available for Germany on excess mortality in diabetes, i.e. the mortality rate in people with diabetes compared to the mortality rate in people without diabetes. These show that excess mortality remains significantly elevated, with no clear indication of a current decline [11, 22, 23]. A decline in excess mortality could be due, for example, to improvements in early detection and earlier treatment of diabetes with fewer complications; an increase, on the other hand, could indicate a deterioration in care.

This article presents forecasts for the temporal development of diagnosed type 2 diabetes in Germany with a time horizon up to 2050. The latest available baseline data is used for mathematical modelling, and various scenarios for the development of incidence and excess mortality are assumed.

**Key messages**

► All scenarios predict rising prevalence and case numbers of type 2 diabetes between 2022 and 2050 in Germany.
► A constant incidence and excess mortality of diabetes (compared to no diabetes) over time results in a prevalence of 16.1 % (number of cases: 11.01 million) in 2050 compared to 8.6 % (6.05 million) in 2022.
► In comparison, a 2.0 % annual decrease in incidence with constant excess mortality leads to a lower increase in prevalence (12.2 % or 8.39 million).
► With a simultaneous decrease in excess mortality of 2.0 % per year, the prevalence increases moderately (13.0 % or 8.94 million).
► In all scenarios, women are less likely to be affected by type 2 diabetes than men.

## 2. Methods

### 2.1 Baseline data

To forecast the development over time until 2050, age-specific data on the prevalence, incidence and excess mortality of diagnosed type 2 diabetes are required as a starting point, as well as estimates of population development until 2050.

The data basis for age-specific prevalence rates of diabetes for people aged 18 and older is the population-based survey GEDA conducted by the Robert Koch Institute [7]. The age-specific prevalence rates for type 2 diabetes are calculated by applying the age-specific proportions of type 2 diabetes cases among all documented diabetes cases, based on a random sample of BARMER insured persons from 2012 to 2018 [2]. The prevalence of documented type 2 diabetes for persons aged 11 to 17 years are drawn from the nationwide Diabetes Prospective Follow-up Registry (DPV) for the year 2022 [24] (Annex Table 1).

The baseline values for age-specific incidence rates of type 2 diabetes, taking into account all age groups, are based on routine data from statutory guild- and company-based health insurance funds for 2021 [16] (Annex Table 2).

Age-specific excess mortality, defined as the ratio of the mortality rate (MR) in people with diabetes to the mortality rate in people without diabetes (mortality rate ratio, MRR), are based on data from 47.3 million persons aged 30 years and over with statutory health insurance for the years 2013/2014 [23], of whom 6.5 million had documented diabetes and 40.8 million had no documented diabetes in 2013. In the following year, 2014, 0.29 million people with diabetes and 0.48 million people without diabetes died. The age-adjusted excess mortality rate was 1.52 for women and 1.56 for men.

Population development indicators, regarding birth rate, life expectancy and net migration between 2022 and 2050, are taken from the 15th Coordinated Population Projection (KoBeV) of the Federal Statistical Office of Germany [25]. For the projection, we considered variant G1L1W2 with a low increase in birth rate (G), a low increase in life expectancy (L) and a net migration (W) of 293,000 people per year (Annex Table 3).

### 2.2 Scenarios for the predicted prevalence and number of cases of diagnosed type 2 diabetes

To project the prevalence and number of cases of diagnosed type 2 diabetes between 2022 and 2050, 14 different scenarios are calculated based on assumptions regarding the development of age-specific incidence and mortality (Table 1).

First, scenarios 1 to 5 from a previous projection by Tönnies et al. (with a time horizon up to 2040) are used [12] and recalculated using more recent baseline values for prevalence, incidence and mortality rate ratio, extending the time horizon to 2050. In scenario 1, the age-specific prevalence rates from 2023 to 2050 correspond to those of the base year 2022. In scenario 2, incidence and excess mortality are assumed to remain constant. In scenarios 3 to 5, the excess mortality changes by - 2.0 % annually, while the incidence is assumed to remain constant (scenario 3) or to change by + 0.5 % annually (scenario 4) or - 0.5 % annually (scenario 5). In addition, in accordance with the information from the literature described in the introduction, further possible scenarios are assumed in the present projections, with annual changes in the incidence rate of -1.0 % and - 2.0 % and in excess mortality of -1.0 %, - 0.5 %, 0 (i.e. constant) and + 0.5 % (scenarios 6 to 13). In a final scenario (scenario 14), an annual change in both excess mortality and incidence of - 2.0 % are assumed. The relative change in the prevalence and case number development, independent of demographic change, is calculated from the quotient of the projected prevalence or case number from scenarios 2 to 14 and the projected prevalence and case number from scenario 1. The projected prevalence or case number is higher than would be expected based on demographic change by this factor.

**Figure 1:**
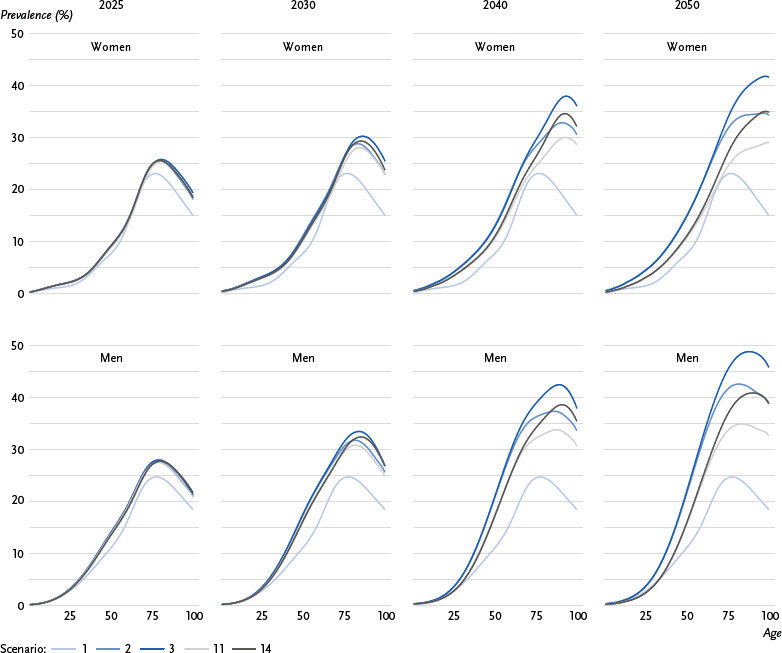
Trends in the prevalence of diagnosed type 2 diabetes in % by age for women and men for the years 2025, 2030, 2040 and 2050 across selected scenarios. Scenario 1: constant prevalence, scenario 2: mortality rate ratio (MRR) 0 %, incidence 0 %, scenario 3: MRR - 2.0 %, incidence 0 %, scenario 11: MRR 0 %, incidence - 2.0 %, scenario 14: MRR - 2.0 %, incidence - 2.0 % Source: Prevalence = GEDA 2022, Diabetes Prospective Follow-up Registry (DPV) data, BARMER data; incidence = data from statutory guild- and company-based health insurance funds; excess mortality = data from all statutory health insurances; population development =15th Coordinated Population Projection (KoBeV)

**Figure 2:**
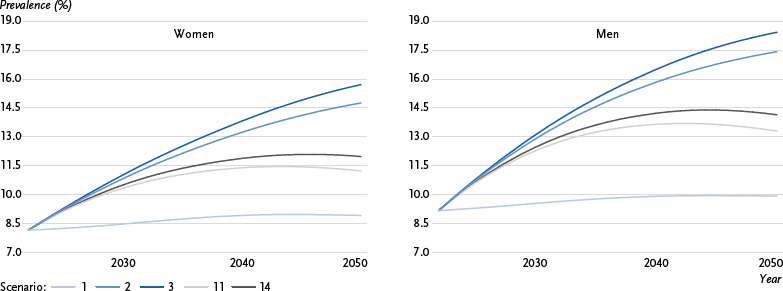
Trends in the overall prevalence of diagnosed type 2 diabetes in % for women and men for the period 2022 to 2050 across selected scenarios. Scenario 1: constant prevalence, scenario 2: mortality rate ratio (MRR) 0 %, incidence 0 %, scenario 3: MRR - 2.0 %, incidence 0 %, scenario 11: MRR 0 %, incidence - 2.0 %, scenario 14: MRR - 2.0 %, incidence - 2.0 %. Source: Prevalence = GEDA 2022, Diabetes Prospective Follow-up Registry (DPV) data, BARMER data; incidence = data from statutory guild- and company-based health insurance funds; excess mortality = data from all statutory health insurances; population development =15th Coordinated Population Projection (KoBeV)

**Figure 3:**
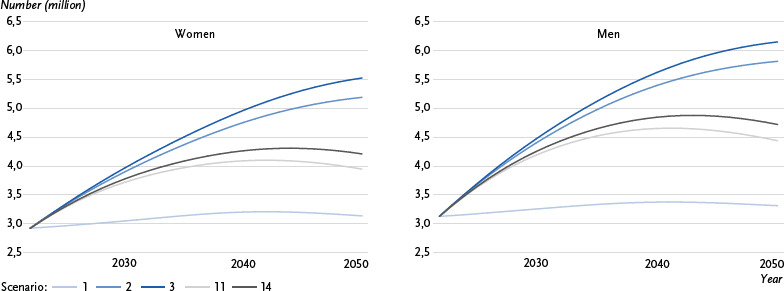
Trends in the number of diagnosed type 2 diabetes cases in millions for women and men in the period 2022 to 2050 across selected scenarios. Scenario 1: constant prevalence, scenario 2: mortality rate ratio (MRR) 0 %, incidence 0 %, scenario 3: MRR - 2.0 %, incidence 0 %, scenario 11: MRR 0 %, incidence - 2.0 %, scenario 14: MRR - 2.0 %, incidence - 2.0 %. Source: Prevalence = GEDA 2022, Diabetes Prospective Follow-up Registry (DPV) data, BARMER data; incidence = data from statutory guild- and company-based health insurance funds; excess mortality = data from all statutory health insurance funds; population development =15th Coordinated Population Projection (KoBeV)

The graphical representation (Figure 1, Figure 2 and Figure 3) focuses on five selected scenarios: Scenario 1 corresponds to the development of type 2 diabetes case numbers that would be expected due to demographic change (ageing population) and was used as the baseline scenario in previous forecast studies, with age-specific prevalence assumed to remain constant. In scenario 2, on the other hand, no changes in the incidence and excess mortality of type 2 diabetes are assumed over the forecast period; in contrast to scenario 1, the declining overall mortality in the population over the forecast period is taken into account. In comparison, scenario 3 assumes a significant decrease in excess mortality (- 2.0 % annually) with constant incidence, while scenario 11 assumes the opposite, i.e. a significant decrease in incidence (- 2.0 % annually) with constant excess mortality. Scenario 14, which assumes a significant decrease in both excess mortality and incidence (- 2.0 % annually), was selected to predict the prevalence and number of cases of type 2 diabetes under conditions desirable from a prevention perspective.

### 2.3 Modelling the predicted prevalence and number of cases of diagnosed type 2 diabetes

Mathematical modelling of the trends in prevalence and number of cases of diagnosed type 2 diabetes up to 2050 was carried out using an illness-death model that uses a partial differential equation to model the relationship between prevalence, incidence and mortality, including excess mortality, as a function of age and year [26]. The prevalence of type 2 diabetes for each age and year is predicted based on baseline values for prevalence and incidence (Annex Table 1. and Annex Table 2) for each age and year up to 2050 by integrating the partial differential equation



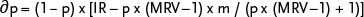



where p = prevalence, IR = incidence, m = total mortality and MRR = mortality rate ratio. Age is included in the model spanning from 0 to 100 years and estimated using spline functions across the specified age groups in the data sources described above (Annex Table 1 and Annex Table 2). The modelling for prevalence and case numbers is carried out separately for women and men; for the total population, the number of cases is calculated as the sum of cases in women and men, and the prevalence is calculated as the number of cases (women + men) / total population (women + men) x 100. The analyses presented here were carried out using the software R, v.4.3.0 (The R Foundation for Statistical Computing).

### 2.4 Sensitivity analyses

In order to compare results based on different assumptions regarding life expectancy and net migration from the 15th KoBeV, additional analyses are performed for other variants as sensitivity analyses (Annex Table 3).

In order to highlight the influence of incidence and excess mortality even more clearly, an additional scenario is calculated with an increase in both incidence and excess mortality (each + 2.0 % per year; scenario 15) that should be avoided from a preventive perspective. In addition, to illustrate the possible range of predicted case numbers, a scenario with a minimum case number (annual change in excess mortality + 2.0 % and incidence - 2.0 %; scenario 16) and a scenario with a maximum case number (annual change in excess mortality - 2.0 % and incidence + 2.0 %; scenario 17) are added.

## 3. Results

### 3.1 Age-specific prevalence of diagnosed type 2 diabetes

Figure 1 shows the predicted prevalence of diagnosed type 2 diabetes among people aged 18 to 100 years for five selected scenarios, based on variant G1L1W2 of the 15th KoBeV, for the years 2025, 2030, 2040 and 2050, and presented separately for women and men. In scenario 1 (assuming constant age-specific prevalence over time), the highest prevalence is expected for people around 80 years of age, with approximately 23 % for women and 25 % for men. In scenario 2 (with constant incidence and excess mortality), a significant increase in age-specific prevalence is expected over time. This increase intensifies with age, shifting the highest prevalence to older age groups. In 2050, the highest prevalence is expected to be approximately 35 % at age 95 for women and 43 % at age 85 for men. In scenario 3 (with constant incidence and a 2.0 % annual decrease in excess mortality), this pattern is expected to be even more pronounced, and the highest prevalence in 2050 is expected to be approximately 42 % for women aged around 95 and 50 % for men aged around 90. In scenario 11 (with constant excess mortality and a 2.0 % annual decrease in incidence), the predicted increase in age-specific prevalence over time is weaker than in scenario 2, and the highest predicted prevalence in 2050 is approximately 30 % for women aged around 100 and 35 % for men aged around 85. In scenario 14 (with incidence and excess mortality decreasing by 2.0 % each), the pattern is expected to be similar to that in scenario 2, but in 2050 the highest age-specific prevalence of approximately 35 % will only be reached at the age of 100 for women and 40 % at the age of 95 for men.

The differences between the individual scenarios increase significantly over time. The higher prevalence rates among men compared to women from the age of 40 onwards can be observed in all five scenarios.

### 3.2 Overall prevalence of diagnosed type 2 diabetes

Figure 2 shows the predicted overall prevalence of type 2 diabetes for the entire age range for the period 2022 to 2050 across the five selected scenarios, presented separately for women and men. The corresponding values for the overall group are shown in Table 2. Assuming constant age-specific prevalence (scenario 1), the prevalence is expected to increase from 8.6 % (women: 8.2 %, men: 9.2 %) in 2022 to 9.4 % (women: 8.9 %, men: 9.9 %) in 2050. In scenarios 2 and 3, the prevalence is predicted to increase to 16.1 % (women: 14.8 %, men: 17.4 %) and 17.0 % (women: 15.7 %, men: 18.4 %), respectively. In scenarios 11 and 14, the prevalence will initially rise over time and then fall again to 12.2 % (women: 11.2 %, men: 13.3 %) and 13.0 % (women: 12.0 %, men: 14.1 %), respectively.

Table 2, which shows all 14 considered scenarios, indicates that the highest increase in the prevalence of diagnosed type 2 diabetes by 2050 is expected to be 18.2 %, assuming an annual increase in incidence by 0.5 % and an annual decline in excess mortality by 2.0 % (scenario 5) (women: 16.9 %, men: 19.7 %). In contrast, apart from scenario 1 (constant age-specific prevalence), the lowest increase in the prevalence of type 2 diabetes is observed assuming an annual decline in incidence by 2.0 % and an annual increase in excess mortality by 0.5 % (scenario 13), leading to a prevalence of 11.9 % in 2050 (women: 10.9 %, men: 13.0 %). In the other calculated scenarios (scenarios 4, 6–10, 12), the predicted prevalence is expected between 12.5 % and 15.9 % (women: 11.5 %–14.7 %, men: 13.6 %–17.2 %) in 2050.

Overall, with decreasing excess mortality and particularly increasing incidence, higher prevalences of diagnosed type 2 diabetes are expected in 2050. For example, a decrease in excess mortality of 2.0 % per year and a change in incidence of - 0.5 %, 0 % or + 0.5 % per year (scenarios 4, 3 and 5) are expected to result in a prevalence of 15.9 %, 17.0 % and 18.2 % (women: 14.7 %, 15.7 %, 16.9 %; men: 17.2 %, 18.4 %, 19.7 %) in 2050. This corresponds to a relative increase in prevalence between 2022 and 2050 of 84 % to 111 % (women: 80 % to 107 %, men: 88 % to 115 %). In comparison, with a decline in incidence of 2.0 % per year and a change in excess mortality of - 0.5 %, 0 % and + 0.5 % per year (scenarios 12, 9 and 13), the prevalence in 2050 is expected to be 13.8 %, 12.5 % and 11.9 % (women: 12.6 %, 11.5 %, 10.9 %; men: 14.9 %, 13.6 %, 13.0 %) in 2050. This corresponds to a relative increase in prevalence of 38 % to 59 % (women: 34 % to 55 %, men: 42 % to 63 %) (Table 2).

Compared to the trend based solely on demographic change (scenario 1), the relative change is lowest in scenario 13, with an increase by a factor of 1.27 (women: 1.22, men: 1.31), and highest in scenario 5, with an increase by a factor of 1.94 (women: 1.90, men: 1.99).

### 3.3 Number of cases of diagnosed type 2 diabetes

Based on the available calculations, the number of people diagnosed with type 2 diabetes in 2022 is expected to be 6.05 million (2.92 million women and 3.13 million men). The projected case numbers across the five selected scenarios for the period 2022 to 2050 are shown, separately for women and for men, in Figure 3, and in Table 2 for the total population. Assuming constant age-specific prevalence (scenario 1), the number of cases is expected to rise to 6.45 million in 2050 (women: 3.14 million, men: 3.32 million). In scenarios 2 and 3, the number of cases is projected to increase to 11.01 million (women: 5.19 million, men: 5.82 million) and 11.68 million (women: 5.53 million, men: 6.15 million), respectively. In scenarios 11 and 14, the number of cases will initially rise over time and then fall again to 8.39 million (women: 3.95 million, men: 4.44 million) and 8.94 million (women: 4.21 million, men: 4.72 million), respectively.

Among all presented scenarios, the projected number of people with type 2 diabetes in 2050 (apart from scenario 1) is lowest in scenario 13 and highest in scenario 5, ranging between 8.18 million and 12.51 million (women: 3.85 million to 5.93 million, men: 4.33 million to 6.58 million). This corresponds to a relative increase in the number of cases of 35.2 % to 106.6 % (women: 31.7 % to 102.8 %, men: 38.4 % to 110.3 %; Table 2).

### 3.4 Sensitivity analyses

In the sensitivity analyses, assuming the three other variants from the 15th KoBeV with regard to life expectancy and net migration (G1L1W1, G1L2W1, G1L2W2), the predicted prevalence and case numbers of diagnosed type 2 diabetes in 2050 are similar to those in the main analysis (using G1L1W2). In the three other variants, for example, the predicted number of cases in 2050 varies between 8.04 million and 8.45 million (women: 3.77 million and 3.98 million, men: 4.27 million and 4.47 million) for scenario 13. For scenario 5, the predicted number of cases ranges between 12.28 million and 12.93 million (women: 5.80 million and 6.13 million, men: 6.48 million and 6.80 million; Annex Table 4).

Assuming a preventable increase in incidence and excess mortality (both + 2.0 % per year; scenario 15), the prevalence of type 2 diabetes is expected to rise to 20.2 % in 2050 (women: 18.6 %, men: 21.8 %). In the scenario with a minimal number of cases (annual change in excess mortality + 2.0 % and incidence - 2.0 %; scenario 16), a prevalence of 10.8 % (women: 9.9 %, men: 11.8 %) is predicted, and in the scenario with a maximum number of cases (annual change in excess mortality - 2.0 % and incidence + 2.0 %; scenario 17), on the other hand, a prevalence of 22.4 % (women: 20.8 %, men: 24.1 %) is predicted. The number of cases of type 2 diabetes in 2050 is estimated at 13.8 million, 7.4 million and 15.3 million in scenarios 15 to 17.

## 4. Discussion

### 4.1 Summary of results

The present work considered a baseline type 2 diabetes prevalence of 8.6 % (women: 8.2 %, men: 9.2 %) and a case number of 6.05 million affected persons (women: 2.92 million, men 3.13 million) in 2022. Assuming constant incidence and excess mortality, the prevalence of diagnosed type 2 diabetes in the German population is expected to increase to 16.1 % (women: 14.8 %, men: 17.4 %) by 2050, corresponding to 11.01 million cases (women: 5.19 million, men: 5.82 million). Assuming a 2.0 % annual decline in incidence, however, the prevalence is expected to increase to 12.2 % (8.39 million) (women: 11.2 % or 3.95 million, men: 13.3 % or 4.44 million). With a simultaneous annual 2.0 % reduction in excess mortality, a prevalence of 13.0 % (8.94 million) is expected (women: 12.0 % or 4.21 million, men 14.1 % or 4.72 million). For women, a smaller increase in prevalence and case numbers is projected between 2022 and 2050 compared to men. This aligns with the absolute and relative differences identified, which are primarily attributable to the higher baseline values in men for prevalence and incidence in almost all age groups.

### 4.2 Comparison of current and previous forecasts

An earlier forecast of the prevalence and case numbers of diagnosed type 2 diabetes identified an increase from 6.9 million cases in 2015 to 10.7 million (+ 54 %) and 12.3 million (+ 77 %) by 2040 as likely scenarios. This forecast assumed an annual change in excess mortality of - 2.0 % and in incidence of - 0.5 % and + 0.5 %, respectively [12]. An update to this forecast [13] incorporated age- and gender-specific incidence trends for the period 2015 to 2021, based on a study using routine data from individuals with statutory health insurance [16], which was also used in this article for the incidence baseline values for the year 2021. This study showed an initial decline in incidence over time for most age groups, followed by a significant increase in incidence in the second pandemic year, 2021 [16]. While scenario 1 of the updated forecast assumed a constant incidence from 2021 to 2040, scenarios 2 to 5 assumed that the observed age- and gender-specific incidence trends between 2015 and 2021 would continue until 2025, 2035 and 2040, respectively, and remain constant thereafter [13]. Using this approach, and assuming an annual change in excess mortality of - 2.0 %, even higher case numbers were projected for 2040, ranging from 10.9 to 14.2 million.

In comparison, the present forecast incorporates a broader range of assumptions regarding possible changes in both incidence and excess mortality in order to illustrate the potential for public health measures. Since there is only limited data available for Germany on excess mortality among people with diabetes compared to people without diabetes, with no clear indication of a current reduction in excess mortality [11, 22, 23], the scenarios include annual changes in excess mortality of - 2.0 % (as in the previous forecasts mentioned) as well as changes of -1.0 %, - 0.5 %, 0 % and + 0.5 %. With regard to incidence, various studies point to a significant decline in the years prior to the pandemic. For example, a study reported an annual decrease in the incidence of type 2 diabetes by approximately 4.9 % in the age group 40 years and older between 2012 and 2014 [27]. Another study found an annual decline of 5.9 % for women and 5.4 % for men in the age group 20 years and older between 2012 and 2017 [17]. A further analysis observed annual reductions across all age groups between 2014/2015 and 2019, ranging from approximately 1.8 % to 2.4 % for women and 0.9 % to 1.7 % for men [16, 28]. However, there is also evidence that the decline in incidence differed during and immediately after the pandemic; i.e. that the overall decline in incidence was less pronounced [17] or that, after an initial sharp decline in 2020, a resurgence in incidence was observed [16, 18]. Therefore, in addition to an annual incidence change of - 2.0 % (pre-pandemic incidence decline), the scenarios in this forecast also consider changes of -1.0 %, 0 %, - 0.5 % and + 0.5 %. Considering the literature and from a prevention perspective, scenario 11, with constant excess mortality rate and a 2.0 % annual decline in incidence, and scenario 14, with a simultaneous 2.0 % annual decline in excess mortality, appear particularly plausible and therefore worth reporting. In both scenarios, the predicted number of cases in 2050 (8.39 million (+ 38.6 %) in scenario 11 and 8.94 million (+ 47.6 %) in scenario 14) are significantly lower than 11.01 million (+ 81.9 %) projected in scenario 2, which assumes constant excess mortality and incidence, and are clearly below the case numbers reported in the earlier forecasts for 2040. Scenario 15 (with a 2.0 % annual increase in both incidence and excess mortality), which was included in the sensitivity analysis and is undesirable from a prevention perspective, results in a significantly higher projected case number of 13.8 million (+128.3 %). The final scenarios included in the sensitivity analysis, scenarios 16 and 17, illustrate the possible range of projections: the lowest estimated number of cases is 7.4 million (+ 22.2 %) with increasing excess mortality and decreasing incidence (both by 2.0 % annually), and the highest estimated number of cases is 15.3 million (+153.5 %) with decreasing excess mortality and increasing incidence (both by 2.0 % annually).

The international Global Burden of Disease study predicts a global increase in the population with diabetes from 529 million in 2021 to 1,310 million in 2050, mainly due to the increase in diabetes prevalence outside Europe [15]. In Germany, the number of people with diabetes is estimated to rise from 7.1 million in 2021 to 10.4 million in 2050 [15]. According to forecasts by the International Diabetes Federation, the prevalence of diabetes in Germany among people aged 20 to 79 is expected to remain relatively constant, with a slight decline from 6.2 million in 2021 to 6.1 million in 2045 [29]. These results indicate that an increase in diabetes cases is mainly attributable to older age groups. This is also supported by the observations on the temporal development of age-specific prevalence rates in this article (Figure 1).

### 4.3 Strengths and limitations

The strength of the present article lies in the use of the illness-death model, which allows assumptions about incidence and mortality trends to be included in modelling the future prevalence and case numbers of type 2 diabetes, resulting in better forecasts [30]. Recent data from 2022 and 2021 were used as baseline values for prevalence and incidence, respectively. The most recent published time series were used for assumptions about incidence trends.

However, this study also has limitations. The data sources used were based on different study designs in terms of populations, survey type and observation period. The choice of the data source also depended on its availability, validity and representativeness. Furthermore, the baseline values for the prevalence of diagnosed type 2 diabetes were not obtained directly, but estimated using an algorithm that combined data on overall diabetes prevalence (GEDA 2022) with the proportion of type 2 diabetes among total diabetes cases (routine data 2018).

Furthermore, all estimates referred exclusively to diagnosed type 2 diabetes and did not include undiagnosed diabetes. Previous studies using laboratory data have shown that although the proportion of the German population with undiagnosed diabetes has declined significantly over the last few decades [11], in 2010, approximately one in five people with diabetes still had undiagnosed diabetes [31].

The baseline values for excess mortality among people with diabetes compared to people without diabetes were based on data from 2013/2014, which are the latest available data for this indicator in Germany. Further information on excess mortality in Germany was published using data from the population-based German National Health Interview and Examination Survey 1998 (GNHIES98) conducted by the Robert Koch Institute (RKI), including mortality follow-up until 2011 [22], and was used in various recently published forecast studies on diabetes prevalence [12, 13]. More up-to-date information on excess mortality will become available once the simplified access to data from the statutory health insurance system (GKV) is implemented through the Health Data Lab at the Federal Institute for Drugs and Medical Devices (BfArM), a measure which is currently in the planning stage. In addition, this study used data on diabetes rather than type 2 diabetes to calculate excess mortality. However, the proportion of people with type 2 diabetes among all people with diabetes in the age groups 50 and older, where most deaths occur, is around 80 % and higher (Annex Table 1).

### 4.4 Conclusion and outlook

All of the scenarios presented here predict rising numbers of people with type 2 diabetes by 2050. This will result in an increasing demand for diabetes care among the population in Germany, combined with higher healthcare expenditure [3, 4]. According to data from a survey on the care situation of people diagnosed with diabetes in 2021/2022, a total of 87.5 % of adults aged 45 and over with type 2 diabetes are dependent on treatment with insulin or other antidiabetic drugs [32]. In addition to type 2 diabetes, almost three-quarters of those affected have high blood pressure, more than a fifth have cardiovascular comorbidities, and more than a quarter have diabetes-specific complications that require intensive monitoring and treatment, such as kidney and eye diseases, neurological disorders and amputations [32].

Along with the growing demand for care, it’s still important to keep improving the quality of care for type 2 diabetes in Germany. In addition to preventing or delaying the onset of complications and comorbidities and the associated premature mortality [23], this also includes, for example, maintaining the quality of life of people with diabetes, which is identified as an overarching treatment goal in the Type 2 Diabetes Care Guideline [33]. Improvements in the quality of care could be achieved, among other things, by improving the self-management of diabetes among those affected and by increasing participation in diabetes education and regular check-ups by doctors [32, 34, 35]. In addition, greater consideration of individual treatment goals and preferences could be achieved in the context of medical treatment [36], e.g. by asking patients about their individual goals in accordance with their life situation, needs, skills and age when drawing up the treatment plan and prioritising these goals repeatedly throughout the course of the disease [33]. Despite the progress observed in the quality of care in the past [37, 38] and the increasing possibilities for the use of modern diabetes technologies (e.g. through sensor-based continuous glucose monitoring [39]), there is still significant potential for improvement in the quality of care for people with type 2 diabetes in Germany [32, 40]. This poses a particular challenge for the German healthcare system, not only in view of the demographic ageing of the population (and the associated increase in multimorbidity), but also in view of the high proportion of doctors approaching retirement (and the resulting need for young doctors) [41, 42]. Improvements in care would lead to a decrease in excess mortality among people with type 2 diabetes compared to people without diabetes, thereby contributing to a further increase in the number of type 2 diabetes cases. This is clearly illustrated in scenario 3, for example, with an assumed decrease in excess mortality of 2.0 % per year compared to scenario 2 with constant excess mortality. Since demographic ageing also contributes to the increase in the number of type 2 diabetes cases (see scenario 1), reducing the incidence of type 2 diabetes is the only way to counteract the rising number of cases.

A comparison of the calculated scenarios shows that the trends in diabetes incidence has by far the greatest influence on the development of case numbers. This highlights the great potential for primary prevention to reduce and even reverse the increase in type 2 diabetes case numbers. For example, in scenario 11, with an assumed annual decrease in incidence of 2.0 % (with excess mortality remaining constant), the projected case numbers rise until around 2040 and then decline again until 2050. Since various studies based on routine data from people with statutory health insurance have observed a decline in incidence in Germany in the last few years before the pandemic – similar to other high-income countries [43] – such a scenario does not seem unrealistic [16, 17, 27, 28]. However, primary prevention measures are needed to achieve a sustainable reduction in incidence. These should not only include education and communication measures to change behaviour at the individual level, but should also take into account preventive health policy measures, such as strengthening tobacco control measures and taxing foods according to their health value [44–46].

## Figures and Tables

**Table 1:** Scenarios depending on the development of incidence and excess mortality. Source: Scenarios 1 – 5 = Tönnies et al. [12], scenarios 6 – 14 = own considerations

Scenario	Change in MRR per year	Change in incidence per year
	%	%
1	Constant age-specific prevalence	
2	0	0
3	- 2.0	0
4	- 2.0	- 0.5
5	- 2.0	+ 0.5
6	-1.0	-1.0
7	-1.0	- 2.0
8	- 0.5	-1.0
9	- 0.5	- 2.0
10	0	-1.0
11	0	- 2.0
12	+ 0.5	-1.0
13	+ 0.5	- 2.0
14	- 2.0	- 2.0

MRR = mortality rate ratio (mortality rate among people with diabetes/mortality rate among people without diabetes)

**Table 2:** Number of cases and prevalence of diagnosed type 2 diabetes in 2022 and 2050 for women and men as well as for the total population across scenarios 1 to 14, based on variant G1L1W2 of the 15th Coordinated Population Projection (KoBeV). Source: Prevalence = GEDA 2022, Diabetes Prospective Follow-up Registry (DPV) data, BARMER data; incidence = data from statutory guild- and company-based health insurance funds; excess mortality = data from all statutory health insurances; population development =15th KoBeV

Gender	Scenario	Change in MRR per year	Change in incidence per year	Number of type 2 diabetes cases in 2022	Number of type 2 diabetes cases in 2050	Absolute difference in number of cases	Relative difference in number of cases	Prevalence of type 2 diabetes in 2022	Prevalence of type 2 diabetes in 2050	Absolute difference in prevalence	Relative difference in prevalence
		%	%	Million	Million	Million	%	%	%	%	%
**Women**	1	Constant prevalence^*^		2.923	3.135	0.212	7.3	8.2	8.9	0.8	9.4
	2	0	0	2.923	5.192	2.269	77.6	8.2	14.8	6.6	81.1
	3	- 2.0	0	2.923	5.527	2.604	89.1	8.2	15.7	7.6	92.8
	4	- 2.0	- 0.5	2.923	5.158	2.235	76.5	8.2	14.7	6.5	79.9
	5	- 2.0	+ 0.5	2.923	5.927	3.004	102.8	8.2	16.9	8.7	106.7
	6	-1.0	-1.0	2.923	4.718	1.795	61.4	8.2	13.4	5.3	64.6
	7	-1.0	- 2.0	2.923	4.118	1.195	40.9	8.2	11.7	3.6	43.6
	8	- 0.5	-1.0	2.923	4.640	1.717	58.7	8.2	13.2	5.0	61.9
	9	- 0.5	- 2.0	2.923	4.041	1.118	38.2	8.2	11.5	3.3	41.0
	10	0	-1.0	2.923	4.544	1.621	55.5	8.2	12.9	4.8	58.5
	11	0	- 2.0	2.923	3.948	1.025	35.1	8.2	11.2	3.1	37.7
	12	+ 0.5	-1.0	2.923	4.443	1.520	52.0	8.2	12.6	4.5	55.0
	13	+ 0.5	- 2.0	2.923	3.850	0.927	31.7	8.2	10.9	2.8	34.3
	14	- 2.0	- 2.0	2.923	4.213	1.290	44.1	8.2	12.0	3.8	47.0
**Men**	1	Constant prevalence^*^		3.130	3.315	0.185	5.9	9.2	9.9	0.8	8.3
	2	0	0	3.130	5.816	2.686	85.8	9.2	17.4	8.2	89.9
	3	- 2.0	0	3.130	6.152	3.022	96.5	9.2	18.4	9.3	100.9
	4	- 2.0	- 0.5	3.130	5.753	2.623	83.8	9.2	17.2	8.1	87.9
	5	- 2.0	+ 0.5	3.130	6.581	3.451	110.3	9.2	19.7	10.5	114.9
	6	-1.0	-1.0	3.130	5.285	2.155	68.8	9.2	15.8	6.7	72.6
	7	-1.0	- 2.0	3.130	4.625	1.495	47.8	9.2	13.9	4.7	51.1
	8	- 0.5	-1.0	3.130	5.201	2.071	66.2	9.2	15.6	6.4	69.8
	9	- 0.5	- 2.0	3.130	4.542	1.412	45.1	9.2	13.6	4.4	48.3
	10	0	-1.0	3.130	5.097	1.967	62.8	9.2	15.3	6.1	66.4
	11	0	- 2.0	3.130	4.439	1.309	41.8	9.2	13.3	4.1	45.0
	12	+ 0.5	-1.0	3.130	4.989	1.859	59.4	9.2	14.9	5.8	62.9
	13	+ 0.5	- 2.0	3.130	4.333	1.203	38.4	9.2	13.0	3.8	41.5
	14	- 2.0	- 2.0	3.130	4.722	1.592	50.9	9.2	14.1	5.0	54.2
**Total**	1	Constant prevalence^*^		6.053	6.451	0.398	6.6	8.6	9.4	0.8	8.8
	2	0	0	6.053	11.008	4.955	81.9	8.6	16.1	7.4	85.7
	3	- 2.0	0	6.053	11.679	5.626	92.9	8.6	17.0	8.4	97.0
	4	- 2.0	- 0.5	6.053	10.911	4.858	80.3	8.6	15.9	7.3	84.0
	5	- 2.0	+ 0.5	6.053	12.508	6.455	106.6	8.6	18.2	9.6	110.9
	6	-1.0	-1.0	6.053	10.003	3.950	65.3	8.6	14.6	5.9	68.7
	7	-1.0	- 2.0	6.053	8.743	2.690	44.4	8.6	12.8	4.1	47.5
	8	- 0.5	-1.0	6.053	9.840	3.787	62.6	8.6	14.4	5.7	66.0
	9	- 0.5	- 2.0	6.053	8.584	2.531	41.8	8.6	12.5	3.9	44.8
	10	0	-1.0	6.053	9.640	3.587	59.3	8.6	14.1	5.4	62.6
	11	0	- 2.0	6.053	8.388	2.335	38.6	8.6	12.2	3.6	41.5
	12	+ 0.5	-1.0	6.053	9.432	3.379	55.8	8.6	13.8	5.1	59.1
	13	+ 0.5	- 2.0	6.053	8.183	2.130	35.2	8.6	11.9	3.3	38.0
	14	- 2.0	- 2.0	6.053	8.935	2.882	47.6	8.6	13.0	4.4	50.7

MRR = mortality rate ratio

^*^age-specific

## Appendix

**Annex Table 1:** Distribution of baseline values for the prevalence of type 2 diabetes in 2022 by age group for women and men. Source: GEDA 2022^*^ [7], Diabetes Prospective Follow-up Registry (DPV) data [47], BARMER data [2]

	Women			Men		
Age (in years)	Prevalence of diabetes	Proportion of type 2 diabetes^**^	Prevalence of type 2 diabetes	Prevalence of diabetes	Proportion of type 2 diabetes^**^	Prevalence of type 2 diabetes
	%		%	%		%
≤10	-	-	0.0	-	-	0.0
11 – 17	-	-	0.0215	-	-	0.0164
18 – 39	1.8	0.5581	1.0047	2.1	0.4187	0.8793
40 – 49	4.1	0.6486	2.6591	9.1	0.5485	4.9917
50 – 59	7.8	0.7964	6.2117	11.5	0.8156	9.3793
60 – 69	12.9	0.9078	11.7102	17.1	0.9053	15.4802
70 – 79	21.8	0.9569	20.8615	24.0	0.9567	22.9604
≥ 80	19.5	0.9771	19.0538	21.8	0.9754	21.2647

^*^Adjusted for age groups

^**^Number of people with type 2 diabetes divided by the number of people with total diabetes

**Annex Table 2:** Distribution of baseline values for the incidence of type 2 diabetes in 2021 by age group for women and men (n = 5,478,769 people at risk). Source: Data from statutory guild- and company-based health insurance funds^*^ [16]

	Women	Men
Age (in years)	Incidence Type 2 diabetes	Incidence Type 2 diabetes
	%	%
≤17	0.0108	0.0140
18 – 34	0.1800	0.1141
35 – 49	0.4296	0.6350
50 – 64	0.9928	1.6072
65 – 79	1.7063	2.2437
≥ 80	1.6741	1.9468

^*^Age groups adjusted

**Annex Table 3:** Selected variants of the 15th Coordinated Population Projection (2021) with assumptions on birth rates, life expectancy and net migration. Source: 15th Coordinated Population Projection (KoBeV) [25]

Variant	Birth rate	Life expectancy in 2070 in years	Net migration: Average number of people per year in the period 2022 – 2070
G1L1W1	Decline until 2032, from 2032: 1.44	Girls: 86.1 Boys: 82.6	183,000
G1L1W2	Decline until 2032, from 2032: 1.44	Girls: 86.1 Boys: 82.6	293,000
G1L2W1	Decline until 2032, from 2032: 1.44	Girls: 88.2 Boys: 84.6	183,000
G1L2W2	Decline until 2032, from 2032: 1.44	Girls: 88.2 Boys: 84.6	293,000

G = birth rate, L = life expectancy, W = net migration

**Annex Table 4:** Number of cases and prevalence of diagnosed type 2 diabetes for 2022 and 2050 for women and men as well as overall for scenarios 1 to 14 for variants G1L1W1, G1L1W2, G1L2W1 and G1L2W2 of the 15th Coordinated Population Projection (KoBeV). Source: Prevalence = GEDA 2022, Diabetes Prospective Follow-up Registry (DPV) data, BARMER data; incidence = data from statutory guild- and company-based health insurance funds; excess mortality = data from all statutory health insurance funds; population development =15th KoBeV

Gender	Scenario	Change MRR per year	Change in incidence per year	Number of type 2 diabetes cases in 2022	Number of cases of type 2 diabetes in 2050 G1L1W1	Number of cases of type 2 diabetes in 2050 G1L1W2	Number of cases of type 2 diabetes in 2050 G1L2W1	Number of cases of type 2 diabetes in 2050 G1L2W2	Prevalence of type 2 diabetes in 2022	Prevalence of type 2 diabetes in 2050 G1L1W1	Prevalence of type 2 diabetes in 2050 G1L1W2	Prevalence of type 2 diabetes in 2050 G1L2W1	Prevalence type 2 diabetes in 2050 G1L2W2
		%	%	Million	Million	Million	Million	Million	%	%	%	%	%
Women	1	Constant prevalence^*^		2.923	3.081	3.135	3.185	3.239	8.2	9.2	8.9	9.4	9.1
	2	0	0	2.923	5.078	5.192	5.246	5.360	8.2	15.2	14.8	15.4	15.0
	3	- 2.0	0	2.923	5.412	5.527	5.603	5.718	8.2	16.2	15.7	16.5	16.0
	4	- 2.0	- 0.5	2.923	5.053	5.158	5.233	5.338	8.2	15.1	14.7	15.4	15.0
	5	- 2.0	+ 0.5	2.923	5.802	5.927	6.003	6.128	8.2	17.4	16.9	17.7	17.2
	6	-1.0	-1.0	2.923	4.622	4.718	4.787	4.884	8.2	13.8	13.4	14.1	13.7
	7	-1.0	- 2.0	2.923	4.036	4.118	4.184	4.266	8.2	12.1	11.7	12.3	11.9
	8	- 0.5	-1.0	2.923	4.544	4.640	4.703	4.800	8.2	13.6	13.2	13.8	13.4
	9	- 0.5	- 2.0	2.923	3.960	4.041	4.103	4.184	8.2	11.8	11.5	12.1	11.7
	10	0	-1.0	2.923	4.448	4.544	4.599	4.695	8.2	13.3	12.9	13.5	13.2
	11	0	- 2.0	2.923	3.867	3.948	4.001	4.083	8.2	11.6	11.2	11.8	11.4
	12	+ 0.5	-1.0	2.923	4.347	4.443	4.489	4.585	8.2	13.0	12.6	13.2	12.8
	13	+ 0.5	- 2.0	2.923	3.769	3.850	3.894	3.976	8.2	11.3	10.9	11.5	11.1
	14	- 2.0	- 2.0	2.923	4.131	4.213	4.285	4.366	8.2	12.4	12.0	12.6	12.2
Men	1	Constant prevalence^*^		3.130	3.258	3.315	3.360	3.418	9.2	10.3	9.9	10.4	10.1
	2	0	0	3.130	5.725	5.816	5.909	6.000	9.2	18.0	17.4	18.3	17.7
	3	- 2.0	0	3.130	6.061	6.152	6.266	6.357	9.2	19.1	18.4	19.4	18.8
	4	- 2.0	- 0.5	3.130	5.670	5.753	5.865	5.947	9.2	17.9	17.2	18.2	17.6
	5	- 2.0	+ 0.5	3.130	6.481	6.581	6.696	6.797	9.2	20.4	19.7	20.8	20.1
	6	-1.0	-1.0	3.130	5.210	5.285	5.390	5.464	9.2	16.4	15.8	16.7	16.1
	7	-1.0	- 2.0	3.130	4.564	4.625	4.725	4.787	9.2	14.4	13.9	14.7	14.1
	8	- 0.5	-1.0	3.130	5.126	5.201	5.300	5.375	9.2	16.1	15.6	16.4	15.9
	9	- 0.5	- 2.0	3.130	4.480	4.542	4.637	4.698	9.2	14.1	13.6	14.4	13.9
	10	0	-1.0	3.130	5.021	5.097	5.188	5.263	9.2	15.8	15.3	16.1	15.5
	11	0	- 2.0	3.130	4.377	4.439	4.526	4.588	9.2	13.8	13.3	14.0	13.5
	12	+ 0.5	-1.0	3.130	4.913	4.989	5.071	5.146	9.2	15.5	14.9	15.7	15.2
	13	+ 0.5	- 2.0	3.130	4.270	4.333	4.410	4.473	9.2	13.4	13.0	13.7	13.2
	14	- 2.0	- 2.0	3.130	4.660	4.722	4.827	4.888	9.2	14.7	14.1	15.0	14.4
Total	1	Constant prevalence^*^		6.053	6.339	6.451	6.545	6.657	8.6	9.7	9.4	9.9	9.6
	2	0	0	6.053	10.803	11.008	11.155	11.360	8.6	16.6	16.1	16.8	16.3
	3	- 2.0	0	6.053	11.473	11.679	11.869	12.075	8.6	17.6	17.0	17.9	17.4
	4	- 2.0	- 0.5	6.053	10.723	10.911	11.098	11.286	8.6	16.4	15.9	16.8	16.2
	5	- 2.0	+ 0.5	6.053	12.282	12.508	12.700	12.925	8.6	18.8	18.2	19.2	18.6
	6	-1.0	-1.0	6.053	9.832	10.003	10.177	10.348	8.6	15.1	14.6	15.4	14.9
	7	-1.0	- 2.0	6.053	8.600	8.743	8.909	9.052	8.6	13.2	12.8	13.5	13.0
	8	- 0.5	-1.0	6.053	9.669	9.840	10.003	10.174	8.6	14.8	14.4	15.1	14.6
	9	- 0.5	- 2.0	6.053	8.440	8.584	8.739	8.882	8.6	12.9	12.5	13.2	12.8
	10	0	-1.0	6.053	9.469	9.640	9.787	9.958	8.6	14.5	14.1	14.8	14.3
	11	0	- 2.0	6.053	8.244	8.388	8.527	8.671	8.6	12.6	12.2	12.9	12.5
	12	+ 0.5	-1.0	6.053	9.260	9.432	9.560	9.731	8.6	14.2	13.8	14.4	14.0
	13	+ 0.5	- 2.0	6.053	8.039	8.183	8.305	8.448	8.6	12.3	11.9	12.5	12.1
	14	- 2.0	- 2.0	6.053	8.791	8.935	9.111	9.255	8.6	13.5	13.0	13.8	13.3

MRR = mortality rate ratio, G = birth rate, L = life expectancy, W = net migration

^*^age-specific
